# Sparsity-Aware DOA Estimation Scheme for Noncircular Source in MIMO Radar

**DOI:** 10.3390/s16040539

**Published:** 2016-04-14

**Authors:** Xianpeng Wang, Wei Wang, Xin Li, Qi Liu, Jing Liu

**Affiliations:** College of Automation, Harbin Engineering University, No. 145 Nantong Street, Harbin 150001, China; wangxianpeng@hrbeu.edu.cn (W.X.); xinxin_forever@126.com (X.L.); lq_skyven@126.com (Q.L.); angleliujing@126.com (J.L.)

**Keywords:** MIMO radar, DOA estimation, noncircular signal, sparse representation, reweighted *l*_1_ norm penalty

## Abstract

In this paper, a novel sparsity-aware direction of arrival (DOA) estimation scheme for a noncircular source is proposed in multiple-input multiple-output (MIMO) radar. In the proposed method, the reduced-dimensional transformation technique is adopted to eliminate the redundant elements. Then, exploiting the noncircularity of signals, a joint sparsity-aware scheme based on the reweighted $l_{1}$ norm penalty is formulated for DOA estimation, in which the diagonal elements of the weight matrix are the coefficients of the noncircular MUSIC-like (NC MUSIC-like) spectrum. Compared to the existing $l_{1}$ norm penalty-based methods, the proposed scheme provides higher angular resolution and better DOA estimation performance. Results from numerical experiments are used to show the effectiveness of our proposed method.

## 1. Introduction

Multiple-input multiple-output (MIMO) radar [1] has been presented as a novel sensor array configuration for several years, and the advantages of MIMO radar have been investigated in [2,3,4,5]. In general, MIMO radar can be categorized into statistical MIMO radar [2] and colocated MIMO radar [3]. The purpose of statistical MIMO radar is to use the separated antennas in transmit and receive arrays to achieve the spatial diversity gain [2]. In contrast, the colocated MIMO radar aims at forming a virtual array with a large aperture by exploiting the orthogonal waveforms emitted by the transmit array, which provides higher angular resolution for DOA estimation [3]. We investigate the DOA estimation in colocated MIMO radar, where the transmit and receive arrays are located closely.

DOA estimation is a key issue in both conventional sensor array signal processing [6,7,8] and MIMO radar [9,10,11,12]. For this issue, subspace-based high-resolution DOA estimation methods, such as multiple signal classification (MUSIC) [9], estimation of signal parameters via rotational invariance technique (ESPRIT) [10] and tensor-based ESPRIT [11], have been investigated for estimating the angles in MIMO radar. These subspace-based methods can obtain satisfying performance with sufficient snapshots and an adequate signal-to-noise ratio (SNR). In addition, a reduced dimensional ESPRIT (RD-ESPRIT) is proposed in [12] by utilizing the reduced dimensional transformation technique, which obtains the similar DOA estimation performance with the ESPRIT algorithm in [10], but has lower computational complexity. Unfortunately, the DOA estimation performance of these subspace-based methods will degrade significantly in the challenged criteria, such as low SNR and limited snapshots. On the other hand, the sparse representation-based (SR-based) signal recover techniques applied for DOA estimation have attracted more and more attention in recent years. In [13], an SR-based method named $l_{1}$-SVD (singular value decomposition) is presented, which converts the DOA estimation into a convex optimization problem by using the spatial sparsity of the source in sensor array systems, and the other sparse DOA estimation methods are investigated via sparse recovery of the covariance matrix or vector in [14,15], respectively. For MIMO radar, a reweighted $l_{1}$-norm penalty method is presented for DOA estimation in [16], in which the reduced dimensional Capon spectrum is used to design the weight matrix for improving the DOA estimation performance. A real-valued version of the sparse representation scheme is proposed for DOA estimation with lower computational complexity in [17], and the SR-based 2D DOA estimation is considered in [18]. The simulation results in [13,14,15,16,17,18] have verified that the SR-based methods exhibit better DOA estimation performance than subspace-based methods with low SNR and limited snapshots.

In general, the DOA estimation precision depends on the aperture of array, and enlarging the aperture of array for improving the estimation precision without extra antennas is an important aspect in DOA estimation. The concept of noncircularity of complex random variables, vectors or signals is introduced in [19], and the noncircularity of signals is usually considered in widely linear processing [20,21]. It has been pointed out that the elliptic covariance matrix is $E(\bar{s}{\bar{s}}^{T})\neq 0$ for noncircular signals, while $E(\bar{s}{\bar{s}}^{T})=0$ for circular signals, where $\bar{s}$ is the complex noncircular signals. In practical communication and radar systems, the complex noncircular signals widely exist, such as binary phase shift keying (BPSK), amplitude modulation (AM) and unbalanced quadrature phase shift keying (UQPSK) modulated signals. The information of the elliptic covariance matrix of these noncircular signals can be used to enlarge the aperture of the array without extra antennas. Then, some subspace-based methods are derived for DOA estimation in MIMO radar [22,23,24], and they provide better DOA estimation performance than traditional subspace-based methods. However, these subspace-based methods [22,23,24] cannot be adapted to the challenging criteria mentioned above. To the best of our knowledge, there is no literature about the SR-based DOA estimation for noncircular signals with an enlarged virtual array in MIMO radar. Thus, we investigate the way to exploit the noncircularity of signals for improving the DOA estimation in MIMO radar by using the sparse representation perspective.

Different from the previous SR-based methods [16,17,18] based on the assumption of complex circular signals, we consider the complex noncircular signals in MIMO radar and propose a novel sparsity-aware DOA estimation scheme for improving the performance. The contributions of the proposed method are summarized as follows: (i) utilize the reduced dimensional transformation matrix to eliminate the redundant elements in MIMO radar, then the received data can be extended by utilizing the noncircularity of signals; (ii) formulate a weight matrix for enhancing the sparsity of the solution by using the MUSIC-like spectrum; (iii) formulate a joint sparsity-aware scheme based on the reweighted $l_{1}$ norm penalty for DOA estimation. Due to using both the the noncircularity of signals and the reweighted $l_{1}$ norm penalty to enhance the sparsity of the solution, the proposed scheme achieves better angle estimation performance and higher resolution than traditional $l_{1}$ norm penalty-based methods.

The remainder of the paper is organized as follows. In Section 2, we describe the MIMO radar signal model with noncircular signals. A joint sparsity-aware scheme based on the reweighted $l_{1}$ norm penalty for DOA estimation of noncircular sources is proposed in Section 3. In Section 4, we give some related remarks and the Cram$\overset{`}{e}$r–Rao bound of noncircular signals in MIMO radar. Simulation results are presented to demonstrate the advantages of the proposed scheme, while the conclusions are given in Section 6.

*Notation*: ${\left(\cdot \right)}^{H}$, ${\left(\cdot \right)}^{T}$, ${\left(\cdot \right)}^{-1}$, ${\left(\cdot \right)}^{*}$ and det(·) are conjugate-transpose, transpose, inverse, conjugate and determinant operators, respectively. ${\left\{A\right\}}^{\left(l_{2}\right)}$ represents a column vector whose *q*-th element is equal to the $l_{2}$ norm of the *q*-th row of A. ⊗ and ⊙ denote the Kronecker product and Khatri–Rao product, respectively; $I_{K}$ is a K×K dimensional unit matrix. E[·] denotes the expectation operator, and diag(a) represents a diagonal matrix whose diagonal elements are the components of the vector a. $\left|\right|\cdot {\left|\right|}_{1}$ and $\left|\right|\cdot {\left|\right|}_{F}$ are the $l_{1}$ norm and Frobenius norm, respectively.

## 2. Problem Formulation

### 2.1. MIMO Radar Signal Model

**Figure 1:**
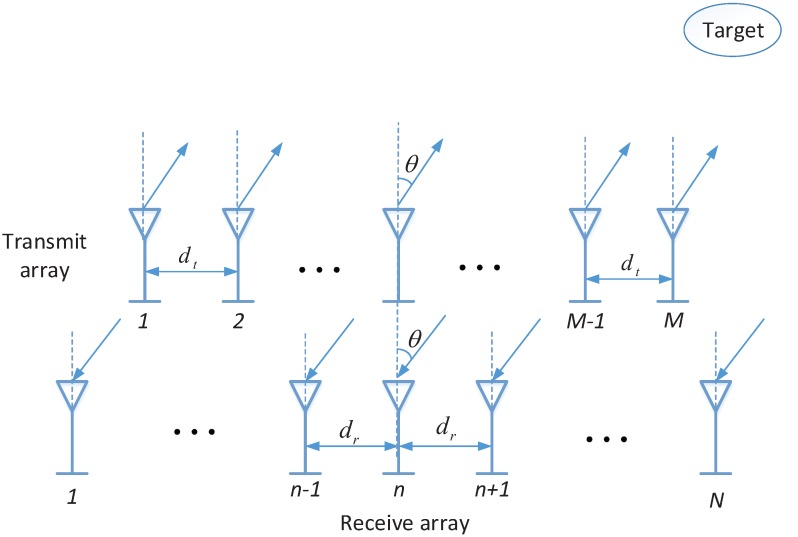
The configuration of the MIMO radar.

We assume a narrowband colocated MIMO radar, shown in Figure 1, with *M* transmit antennas and *N* receive antennas. In such system, both transmit and receive antennas are arranged in half-wavelength-spaced uniform linear arrays (ULAs), *i.e.*, $d_{r}=d_{t}=\lambda /2$, where $d_{r}$ and $d_{t}$ are the distance between adjacent sensors in the transmit and receive arrays, respectively, and *λ* is the wavelength. The transmit and receive arrays are located closely; therefore, the direction of arrivals (DOAs) of a target with respect to both transmit and receive arrays can be regarded as essentially the same. In the transmit side, *M* orthogonal noncircular waveforms, supposed BPSK modulation, are emitted, and the matched filters are designed according to these orthogonal waveforms. Let *P* denote the number of targets, and it is assumed to be known in this paper. The received data obtained from the receive antennas can be processed by utilizing matched filters, then the received data can be modeled as [16,17]:

(1) $$x\left(t\right)=As_{c}\left(t\right)+n\left(t\right)$$

where $x\left(t\right)\in C^{MN\times 1}$ is the received data vector and $s_{c}\left(t\right)\in C^{P\times 1}$ is the complex-valued noncircular signal vector. $A=A_{t}⊙A_{r}=\left[a_{t}\left(\theta_{1}\right)⊗a_{r}\left(\theta_{1}\right),\cdots ,a_{t}\left(\theta_{P}\right)⊗a_{r}\left(\theta_{P}\right)\right]\in C^{MN\times P}$ is the transmit-receive steering matrix; $A_{t}=\left[a_{t}\left(\theta_{1}\right)\cdots ,a_{t}\left(\theta_{P}\right)\right]\in C^{M\times P}$ is the transmit steering matrix whose *p*-th column is the transmit steering vector $a_{t}\left(\theta_{p}\right)={\left[1,e^{j\sin\theta_{p}},\cdots ,e^{j\left(M-1\right)\sin\theta_{p}}\right]}^{T},p=1,2,\cdots ,P$ corresponding to the *p*-th DOA denoted as $\theta_{p}$. $A_{r}=\left[a_{r}\left(\theta_{1}\right),\cdots ,a_{r}\left(\theta_{P}\right)\right]\in C^{N\times P}$ is the receive steering matrix composed of the the receive steering vector $a_{r}\left(\theta_{p}\right)={\left[1,e^{j\sin\theta_{p}},\cdots ,e^{j\left(N-1\right)\sin\theta_{p}}\right]}^{T}$. $n\left(t\right)\in C^{MN\times 1}$ is the additional stochastic complex Gaussian noise vector, whose mean and covariance matrix are zeros and $\sigma^{2}I_{MN}$, respectively, and $\sigma^{2}$ is the power of noise. In a practical situation, multiple snapshots are necessary, then the received data matrix can be expressed as:

(2) $$X=AS_{c}+N$$

where $X=\left[x\left(t_{1}\right),x\left(t_{2}\right),\cdots ,x\left(t_{J}\right)\right]\in C^{MN\times J}$ is the received data matrix and *J* is the number of snapshots. $S_{c}=\left[s_{c}\left(t_{1}\right),s_{c}\left(t_{2}\right),\cdots ,s_{c}\left(t_{J}\right)\right]\in C^{P\times J}$ is the noncircular signal matrix. $N=\left[n_{1}\left(t\right),n\left(t_{2}\right),\cdots ,n\left(t_{J}\right)\right]\in C^{MN\times J}$ is the additional stochastic complex Gaussian white noise matrix.

### 2.2. Noncircular Signals

Circularity is a very important characteristic of complex random variables, vectors or signals [19]. For a zero-mean stationary complex signal sequence $y\in C^{m\times 1}$, the concept of circularity based on second orders statistical is defined as:

(3) $$\begin{array}{cc} & E\left[yy^{H}\right]=\delta^{2}\\ & E\left[yy^{T}\right]=\rho e^{\phi }\delta^{2} \end{array}$$

where $e^{\phi }$ is the noncircular phase. 0≤ρ≤1 is the circularity rate depending on the modulation type of the signal. The zero-mean stationary complex signal sequence can be said to be circular if ρ=0 and noncircular if 0<ρ≤1. In this paper, we consider the special modulated signals that are completely noncircular, *i.e.*, ρ=1, like BPSK and AM modulation signals, in MIMO radar. Then, the noncircular signal vector in Equation (1) is expressed as [22,23,24]:

(4) $$\begin{array}{c} s_{c}\left(t\right)=\Phi s\left(t\right) \end{array}$$

where $\Phi =\mathrm{diag}(\left[e^{j\varphi_{1}},\cdots ,e^{j\varphi_{P}}\right])$ is the noncircular phase matrix with the initial phase φ that should be different for each target and satisfies φ=2ϕ when ρ=1 and $s\left(t\right)\in R^{P\times 1}$ is the real-valued part. Obviously, the covariance matrix and elliptic covariance matrix satisfy $E[S_{c}S_{c}^{H}]\neq 0$ and $E[S_{c}S_{c}^{T}]\neq 0$, respectively. Substituting Equation (4) into Equation (2), we have:

(5) X=AΦS+N

where $S=\left[s\left(t_{1}\right),s\left(t_{2}\right),\cdots ,s\left(t_{J}\right)\right]\in R^{P\times J}$ is the real-valued matrix.

## 3. Sparse Representation Scheme for DOA Estimation of a Noncircular Source

In the subspace-based methods [22,23,24], the noncircularity of signals in Equation (5) can be used to enlarge the virtual aperture of MIMO radar, but these subspace-based methods need a large quantity of snapshots and high SNR to obtain high DOA resolution. One the other hand, the sparse representation scheme [16,17] can be straightforwardly applied for DOA estimation based on Equation (2). However, they do not take the possible noncircularity of signals into account. In the following section, a novel joint sparsity-aware scheme based on the reweighted $l_{1}$ norm penalty is formulated for DOA estimation by using the noncircularity of signals.

According to the transmit-receive steering vector $a_{t}\left(\theta \right)⊗a_{r}\left(\theta \right)$, it can be concluded that there exists many redundant elements due to the special configuration of MIMO radar. Then, the steering vector $a_{t}\left(\theta \right)⊗a_{r}\left(\theta \right)$ can be written as:

(6) $$a_{t}\left(\theta \right)⊗a_{r}\left(\theta \right)=Gb\left(\theta \right)$$

where $G\in C^{MN\times Q}$ is a full-rank transformation matrix and $b\left(\theta \right)\in C^{Q\times 1}\left(Q=M+N-1\right)$ is a one-dimensional steering vector. They are expressed as:

(7) $$G={\left[J_{0}^{T},\cdots ,J_{M-1}^{T}\right]}^{T}$$

(8) $$b\left(\theta \right)={\left[1,e^{j\sin\theta_{p}},\cdots ,e^{j\left(Q-1\right)\sin\theta_{p}}\right]}^{T}$$

where $J_{m}=\left[0_{N\times m},I_{N},0_{N\times (M-m-1)}\right],\;m=0,1,...,M-1$. According to the structure of the transformation matrix, the matrix $F=G^{H}G$ is written as:

(9) $$F=\mathrm{diag}[1,2,...,\underset{|M-N|+1}{\underset{︸}{\min(M,N),...,\min(M,N)}},....,2,1]$$

According to Equations (6) and (9), it is indicated that the redundant elements can be eliminated by using the transformation matrix $G^{H}$. However, the colored noise will be added by using $G^{H}$. In order to solve this issue, a novel reduced-dimensional matrix can be formulated as $\bar{G}=F^{-1/2}G^{H}$ [12], which satisfies with $\bar{G}{\bar{G}}^{H}=I_{Q}$, and this means that the matrix $\bar{G}$ is an orthogonal matrix. Then, multiplying $\bar{G}$ by the received data X, we have:

(10) $$\begin{array}{cc} Y & =\bar{G}X=\bar{G}AS_{c}+\bar{G}N\\ & =F^{1/2}BS_{c}+\bar{G}N=\bar{B}S_{c}+\bar{N} \end{array}$$

where $\bar{B}=F^{1/2}B$ with $B=[b\left(\theta_{1}\right),\cdots ,b\left(\theta_{P}\right)]$, and $\bar{N}=\bar{G}N$. After the reduced dimensional transformation, the mean and covariance matrix of the nose matrix are $E\left[\bar{N}\right]=\bar{G}E\left[N\right]=0$ and $E\left[\bar{N}{\bar{N}}^{H}\right]=\bar{G}E\left[NN^{H}\right]{\bar{G}}^{H}=\sigma^{2}I_{Q}$. Thus, the noise matrix is also a complex Gaussian distribution. After eliminating the redundant elements, the matrix Y in Equation (10) can be regarded as the received data of a ULA with weight matrix $F^{1/2}$. In addition, taking the noncircularity of signals into account, *i.e.*, exploiting the information of elliptic covariance matrix $E[S_{c}S_{c}^{T}]\neq 0$, the extended data of Equation (10) can be expressed as [22,23,24]:

(11) $$Z=\left[\begin{array}{c} \Gamma_{Q}Y^{*}\\ Y \end{array}\right]=\left[\begin{array}{c} \Gamma_{Q}{\bar{B}}^{*}S_{c}^{*}\\ \bar{B}S_{c} \end{array}\right]+\left[\begin{array}{c} \Gamma_{Q}{\bar{N}}^{*}\\ \bar{N} \end{array}\right]$$

where $\Gamma_{Q}$ is the Q×Q exchange matrix with ones on its anti-diagonal and zeros elsewhere. Due to the fact that the noncircular signal matrix $S_{c}$ has the real-part $S=S^{*}$ shown in Equation (5), the Equation (11) can be rewritten as:

(12) $$Z=\left[\begin{array}{c} \Gamma_{Q}{\bar{B}}^{*}\Phi^{*}\\ \bar{B}\Phi \end{array}\right]S+\left[\begin{array}{c} \Gamma_{Q}{\bar{N}}^{*}\\ \bar{N} \end{array}\right]=\bar{F}B_{e}S+N_{e}$$

where $N_{e}$ is the noise matrix after extending the received data. $B_{e}=\left[b_{e}\left(\theta_{1},\varphi_{1}\right),\cdots ,b_{e}\left(\theta_{P},\varphi_{P}\right)\right]$ is the new steering matrix, and the steering vector $b_{e}\left(\theta_{p},\varphi_{p}\right)$ and $\bar{F}$ can be written as:

(13) $$\begin{array}{cc} b_{e}\left(\theta_{p},\varphi_{p}\right) & =[e^{-j\left(Q-1\right)\sin\theta_{p}}e^{-j\varphi_{p}},\cdots ,e^{-j\varphi_{p}},e^{j\varphi_{P}},\\ & e^{j\sin\theta_{p}},\cdots ,e^{j\left(Q-1\right)\sin\theta_{p}}e^{j\varphi_{P}}{]}^{T} \end{array}$$

and:

(14) $$\begin{array}{c} \bar{F}=\left[\begin{array}{c} F^{1/2}\;0\\ 0\;F^{1/2} \end{array}\right] \end{array}$$

In order to exploit the sparse representation viewpoint for Equation (12), the sparse signal model must be established firstly by formulating the complete dictionary. Noting the steering vector $b_{e}\left(\theta_{p},\varphi_{p}\right)$ in Equation (13) contains the unknown additional phase, the extended received data Z cannot be turned into the sparse representation model. However, due to the fact that the extended data are composed of the received data in Equation (5) and its conjugation, the extended data Z can be turned into two sparse representation models without the effect of the unknown additional phase, and then, a joint sparsity-aware scheme is proposed for taking the noncircularity into account. When utilizing the sparse representation viewpoint, the SVD technique [13] can be used to reduce the dimension of the recovered matrix. Then, the SVD of the extended received data in Equation (12) can be shown as:

(15) $$\begin{array}{c} Z=\left[U_{s}\;U_{n}\right]\left[\begin{array}{c} \Sigma_{s}\;0\\ 0\;\Sigma_{n} \end{array}\right]\left[\begin{array}{c} V_{s}^{H}\\ V_{n}^{H} \end{array}\right] \end{array}$$

where $U_{s}\in C^{2Q\times P}$ and $V_{s}\in C^{J\times P}$ are composed of left and right singular vectors corresponding to the *P* largest singular values. $U_{n}\in C^{2Q\times (2Q-P)}$ and $V_{n}\in C^{J\times (J-P)}$ are composed of left and right singular vectors corresponding to the residual 2Q-P singular values. $\Sigma_{s}$ and $\Sigma_{n}$ are diagonal matrices whose diagonal elements are the *P* largest singular values and the residual 2Q-P singular values, respectively. Multiplying the received signal Z by $V_{s}$, we can obtain:

(16) $$\left[\begin{array}{c} Z_{s1}\\ Z_{s2} \end{array}\right]=\left[\begin{array}{c} \Gamma_{Q}{\bar{B}}^{*}\Phi^{*}SV_{s}\\ \bar{B}\Phi SV_{s} \end{array}\right]+\left[\begin{array}{c} \Gamma_{Q}{\bar{N}}^{*}V_{s}\\ \bar{N}V_{s} \end{array}\right]=\left[\begin{array}{c} \hat{B}{\hat{S}}_{s}\\ \bar{B}{\bar{S}}_{s} \end{array}\right]+\left[\begin{array}{c} {\hat{N}}_{s}\\ {\bar{N}}_{s} \end{array}\right]$$

where $Z_{s1}\in C^{Q\times P}$ and $Z_{s2}\in C^{Q\times P}$, $\hat{B}=\Gamma_{Q}{\bar{B}}^{*}$, ${\hat{S}}_{s}=\Phi^{*}SV_{s}$, ${\bar{S}}_{s}=\Phi SV_{s}$, ${\hat{N}}_{s}=\Gamma_{Q}{\bar{N}}^{*}V_{s}$ and ${\bar{N}}_{s}=\bar{N}V_{s}$. By exploiting the sparsity of targets corresponding to the whole spatial space, let ${\left\{{\hat{\theta }}_{i}\right\}}_{i=1}^{L}\left(L\gg P\right)$ be a grid that covers Ω, where Ω denotes the set of possible DOAs, and two complete dictionaries can be constructed as:

(17) $$\begin{array}{c} {\bar{B}}_{\hat{\theta }}=F^{1/2}\left[b\left({\hat{\theta }}_{1}\right),b\left({\hat{\theta }}_{2}\right),\cdots ,b\left({\hat{\theta }}_{L}\right)\right]\in C^{Q\times L}\\ {\hat{B}}_{\hat{\theta }}=\Gamma_{Q}F^{1/2}\left[b^{*}\left({\hat{\theta }}_{1}\right),b*\left({\hat{\theta }}_{2}\right),\cdots ,b^{*}\left({\hat{\theta }}_{L}\right)\right]\in C^{Q\times L} \end{array}$$

Then, two sparse representation models corresponding to Equation (16) can be expressed as:

(18) $$\begin{array}{c} Z_{s1}={\hat{B}}_{\hat{\theta }}{\hat{S}}_{s}^{\hat{\theta }}+{\hat{N}}_{s}\\ Z_{s2}={\bar{B}}_{\hat{\theta }}{\bar{S}}_{s}^{\hat{\theta }}+{\bar{N}}_{s} \end{array}$$

where ${\hat{S}}_{s}^{\hat{\theta }}\in C^{L\times P}$ and ${\bar{S}}_{s}^{\hat{\theta }}\in C^{L\times P}$ have the same sparsity with $\Phi^{*}SV_{s}$ and $\Phi SV_{s}$, respectively. The noncircularity of signals cannot be used if the sparse matrices in Equation (18) are solved independently by using the conventional $l_{1}$ norm penalty-based methods [16,17]. In order to use the possible noncircularity of signals, a joint sparsity-aware scheme is proposed for combining the signal information contained in $Z_{s1}$ and $Z_{s2}$. Noting ${\hat{S}}_{s}^{\hat{\theta }}$ and ${\bar{S}}_{s}^{\hat{\theta }}$ have the same sparsity, a joint sparse representation framework based on the $l_{1}$ norm penalty can be formulated for DOA estimation, which can be expressed as:

(19) $$\begin{array}{cc} & \min∥\Upsilon {∥}_{1}\\ & s.t.\;\Upsilon \left(i\right)\geq \sqrt{{\left({\left\{{\bar{S}}_{s}^{\hat{\theta }}\right\}}^{\left(l_{2}\right)}\left(i\right)\right)}^{2}+{\left({\left\{{\hat{S}}_{s}^{\hat{\theta }}\right\}}^{\left(l_{2}\right)}\left(i\right)\right)}^{2}},\\ & \;i=1,2,\cdots ,L.\\ & ∥Z_{s1}-{\hat{B}}_{\hat{\theta }}{\hat{S}}_{s}^{\hat{\theta }}{∥}_{F}\leq \beta_{1}\\ & ∥Z_{s2}-{\bar{B}}_{\hat{\theta }}{\bar{S}}_{s}^{\hat{\theta }}{∥}_{F}\leq \beta_{2} \end{array}$$

where $\Upsilon \in C^{L\times 1}$ is a sparse vector and Υ(i) is the *i*-th element of **Υ**. ${\left\{{\bar{S}}_{s}^{\hat{\theta }}\right\}}^{\left(l_{2}\right)}\left(i\right)$ and ${\left\{{\hat{S}}_{s}^{\hat{\theta }}\right\}}^{\left(l_{2}\right)}\left(i\right)$ are the *i*-th element of ${\left\{{\bar{S}}_{s}^{\hat{\theta }}\right\}}^{\left(l_{2}\right)}$ and ${\left\{{\hat{S}}_{s}^{\hat{\theta }}\right\}}^{\left(l_{2}\right)}$, respectively. $\beta_{1}$ and $\beta_{2}$ are the regularization parameters. It has been pointed out in [25] that the sparse solution of the $l_{1}$-norm penalty does not approximate better to the $l_{0}$-norm penalty in Equation (19), which leads to the limited recover performance. In [16], the weight matrix based on reduced dimensional capon spectrum is designed to enhance the sparsity of the solution. However, it cannot be used to formulate the weight matrix based on Equation (12) due to the unknown additional phase. Inspired by [16,25], a weight matrix can be formulated for enhancing the sparsity of the solution based on the noncircular MUSIC-like spectrum. Exploiting the orthogonality of the steering vector $b_{e}\left(\theta ,\varphi \right)$ and its corresponding noise subspace $U_{n}$, we have:

(20) $$\begin{array}{c} f\left(\theta ,\varphi \right)=\left[e^{j\varphi },e^{-j\varphi }\right]\Omega \left(\theta \right)\left[\begin{array}{c} e^{-j\varphi }\\ e^{j\varphi } \end{array}\right]\to 0 \end{array}$$

where:

(21) $$\begin{array}{cc} \Omega (\theta ) & ={\left[\begin{array}{c} \Gamma_{Q}b^{*}\left(\theta \right)\;0\\ 0\;b(\theta ) \end{array}\right]}^{H}{\bar{F}}^{H}U_{n}U_{n}^{H}\bar{F}\left[\begin{array}{c} \Gamma_{Q}b^{*}\left(\theta \right)\;0\\ 0\;b(\theta ) \end{array}\right]\\ & =C{\left(\theta \right)}^{H}U_{n}U_{n}^{H}C\left(\theta \right) \end{array}$$

and:

(22) $$\begin{array}{c} C\left(\theta \right)=\bar{F}\left[\begin{array}{c} \Gamma_{Q}b^{*}\left(\theta \right)\;0\\ 0\;b(\theta ) \end{array}\right] \end{array}$$

Due to $[e^{j\varphi },e^{-j\varphi }]\neq 0$, the $\Omega \left(\theta \right)\in C^{2\times 2}$ is positive definite and a consistent estimate of the rank deficient matrix. Consequently, the noncircular MUSIC-Like spectrum [26] can be expressed as:

(23) $$\begin{array}{c} f\left(\theta \right)=\arg\min_{\theta }\left(\det\left(\Omega \left(\theta \right)\right)\right) \end{array}$$

According to the grid ${\left\{{\hat{\theta }}_{i}\right\}}_{i=1}^{L}\left(L\gg P\right)$, the matrix $C\left({\hat{\theta }}_{i}\right)\left(i=1,2,\cdots ,L\right)$ can be obtained. Then, the elements of the weight vector $\phi =[\phi_{1},\phi_{2},\cdots ,\phi_{L}]$ can be expressed as:

(24) $$\begin{array}{c} \phi_{i}=\det\left(\Omega \left({\hat{\theta }}_{i}\right)\right),\;i=1,2,\cdots ,L \end{array}$$

It can be concluded that the element $\phi_{i}$ satisfies $\phi_{i}\to 0$ when the DOA ${\hat{\theta }}_{i}$ corresponds to the possible targets and otherwise $\phi_{i}\gg 0$. Then, the weight matrix is formulated as:

(25) W=diag(ϕ)/max(ϕ)

Due to the characteristic that the elements $\phi_{i}/\max\left(\phi \right)\left(i=1,2,\cdots ,P\right)$ corresponding to the possible targets are much smaller than other elements $\phi_{i}/\max\left(\phi \right)\left(i=1,2,\cdots ,L-P\right)$ in Equation (25), the weight matrix W for the MMVproblem can achieve the viewpoint of the reweighted $l_{1}$ norm penalty in [16,25]. Finally, the joint sparsity-aware scheme based on the reweighted $l_{1}$ norm penalty is formulated as:

(26) $$\begin{array}{cc} & \min∥W\Upsilon {∥}_{1}\\ & s.t.\;\Upsilon \left(i\right)\geq \sqrt{{\left({\left\{{\bar{S}}_{s}^{\hat{\theta }}\right\}}^{\left(l_{2}\right)}\left(i\right)\right)}^{2}+{\left({\left\{{\hat{S}}_{s}^{\hat{\theta }}\right\}}^{\left(l_{2}\right)}\left(i\right)\right)}^{2}},\\ & \;i=1,2,\cdots ,L.\\ & ∥Z_{s1}-{\hat{B}}_{\hat{\theta }}{\hat{S}}_{s}^{\hat{\theta }}{∥}_{F}\leq \beta_{1}\\ & ∥Z_{s2}-{\bar{B}}_{\hat{\theta }}{\bar{S}}_{s}^{\hat{\theta }}{∥}_{F}\leq \beta_{2} \end{array}$$

Obviously, Equation (26) is a convex optimization problem, and some SOC (second order cone) programming software packages, such as CVX [27] and SeDuMi [28], can be used to solve it effectively. Then, the DOAs of targets are estimated by searching the spectrum of **Υ**.

## 4. Related Remarks and the Cramer–Rao Bound

*Remark 1:* In Equations (19) and (26), the selection of the regularization parameters $\beta_{1}$ and $\beta_{2}$ is very important for final DOA estimation and depends on the distribution of the noise matrices ${\hat{N}}_{s}$ and ${\bar{N}}_{s}$. Due to the fact that both $\left|\right|{\hat{N}}_{s}{\left|\right|}_{F}^{2}$ and $\left|\right|{\bar{N}}_{s}{\left|\right|}_{F}^{2}$ satisfy with the asymptotically chi-square distributed with QP degrees of freedom, the parameters $\beta_{1}$ and $\beta_{2}$ can be selected as the upper value of $\left|\right|{\hat{N}}_{s}{\left|\right|}_{F}^{2}$ and $\left|\right|{\bar{N}}_{s}{\left|\right|}_{F}^{2}$ with a high probability 1-ξ confidence interval, *i.e.*, ξ=0.01 is enough.

*Remark 2:* Compared to the conventional $l_{1}$-norm penalty-based methods, the proposed sparsity-aware scheme uses the noncircularity of signals to improve the performance. Furthermore, due to the NC MUSIC-like spectrum corresponding to the larger virtual aperture in MIMO radar, the weight matrix can be formulated more correctly to punish the entries. Consequently, the enhanced sparse solution can be obtained, and then, the angle estimation performance can be further improved.

*Remark 3:* The computational complexity of the proposed method is analyzed and compared to the $l_{1}$-SVD method [13] and the reweighted $l_{1}$-norm penalty method in [16]. According to the implementation procedure of the proposed method, the main computational burden of the proposed method focuses on designing the weight matrix and solving the convex optimization problem in Equation (26). Designing the weighted matrix requires $O\{8Q^{3}+\left(8Q^{3}+\left(8-4P\right)Q^{2}+8Q\right)L\}$, and solving the convex optimization problem requires $O\{LP^{3}\}$. Then, the total computational complexity of the proposed method is $O\{\left(8Q^{3}+\left[8Q^{3}+\left(8-4P\right)Q^{2}+8Q\right]L+LP^{3}\right\}$; while the $l_{1}$-SVD and reweighted $l_{1}$-norm penalty methods require $O\{LP^{3}\}$ and $O\{Q^{2}J+Q^{3}+L\left[Q^{2}+\left(P-1\right)Q-P\right]+LP^{3}\}$, respectively. The proposed method has higher computational complexity than both the $l_{1}$-SVD and the reweighted $l_{1}$-norm penalty methods, but the proposed method achieves better performance and higher resolution than them.

*Cramer–Rao bound (CRB):* In this section, we derive the CRB of DOA estimation for noncircular signals in MIMO radar. Exploiting the noncircularity of the signals, the received data in Equation (2) can be extended as [22,23,24]:

(27) $$\bar{Y}=\left[\begin{array}{c} \Gamma_{MN}X^{*}\\ X \end{array}\right]=\left[\begin{array}{c} \Gamma_{MN}A^{*}s_{c}^{*}\left(t\right)\\ As_{c}\left(t\right) \end{array}\right]+\left[\begin{array}{c} \Gamma_{MN}N^{*}\\ N \end{array}\right]=\bar{A}S+\underline{N}$$

where:

(28) $$\bar{A}=\left[\begin{array}{c} \Gamma_{MN}A^{*}\Phi^{*}\\ A\Phi \end{array}\right]\;\underline{N}=\left[\begin{array}{c} \Gamma_{MN}N^{*}\\ N \end{array}\right]$$

Based on the extended data model in Equation (28), the data model can be regarded as the received data of a novel uniform linear array with the the steering matrix $\bar{A}$, and both the signal matrix S and noise matrix $\underline{N}$ are Gaussian distributed with zero mean. Thus, according to [29], the CRB of DOA estimation for noncircular signals in MIMO radar can be derived as:

(29) CRB=δ22J{Re(DHΠA¯⊥D⊙Ps}-1

where $\delta^{2}$ is the power of the noise. $\Pi_{\bar{A}}^{⊥}=I_{2MN}-\bar{A}{\left({\bar{A}}^{H}\bar{A}\right)}^{-1}{\bar{A}}^{H}$, $P_{s}=E[SS^{H}$] and $D=[\partial {\bar{a}}_{1}\left(\theta \right)/\partial \left(\theta \right),\cdots ,\partial {\bar{a}}_{P}\left(\theta \right)/\partial \left(\theta \right)]$ is the matrix whose *p*-th column is given by the derivative of ${\bar{a}}_{p}\left(\theta \right)$ with respect to $\theta_{p}$. ${\bar{a}}_{p}\left(\theta \right)$ denotes the *p*-th column of $\bar{A}$.

## 5. Simulation Results

In what follows, we evaluate the performance of the proposed method by comparing to the $l_{1}$-SVD method [13], the reweighted $l_{1}$-norm penalty method in [16] and the (CRB) in Equation (29). Except as otherwise noted in the following simulation results, we consider a colocated MIMO radar with *M* = 5 transmit antennas and *N* = 6 receive antennas. Both transmit and receive antennas are arranged in half-wavelength-spaced uniform linear arrays (ULAs), and the transmit and receive arrays are located closely. It is assumed that the number of targets is to be known, and the SNR can be defined as 10log${}_{10}(||AS_{c}{\left|\right|}_{F}^{2}/{\left||N|\right|}_{F}^{2})$. The spatial sampling interval is 0.1° for constructing the complete dictionary, and the confidence interval is set to 0.99 for all of the methods. The root-mean-square-error (RMSE) of the DOA estimation is defined as:

(30) $$\begin{array}{c} \mathrm{RMSE}=\sqrt{\frac{1}{100P}\sum_{i=1}^{100}\sum_{p=1}^{P}{\left({\hat{\theta }}_{i,p}-\theta_{p}\right)}^{2}} \end{array}$$

where ${\hat{\theta }}_{i,p}$ represents the estimation of $\theta_{p}$ at the *i*-th trial.

**Figure 2:**
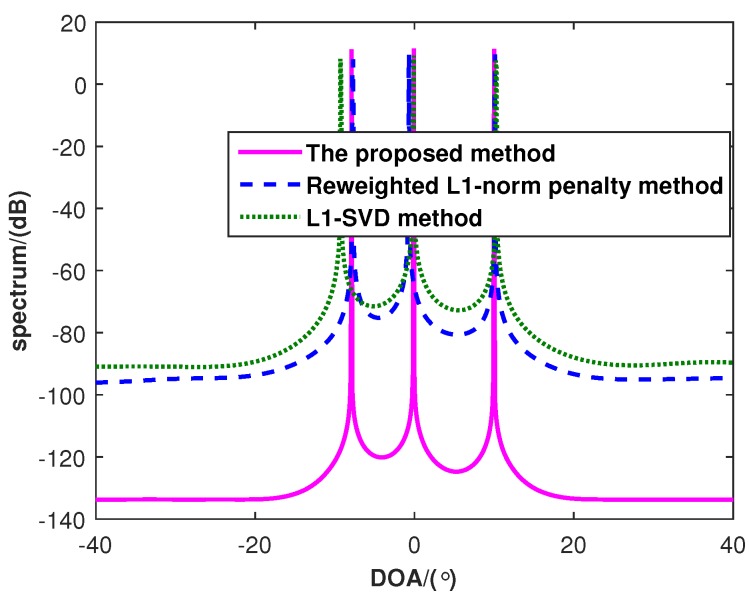
The spatial spectra of the $l_{1}$-SVD method, the reweighted $l_{1}$-norm penalty method and the proposed method.

Figure 2 shows the spatial spectra of three algorithms, where there are P=3 uncorrelated targets with the DOAs as $\theta_{1}=-8$°, $\theta_{2}=0$° and $\theta_{3}=10$°. The SNR is 0 dB, and the number of snapshots is 100. From Figure 2, it is indicated that the proposed method has a lower sidelobe than both the $l_{1}$-SVD algorithm and the reweighted $l_{1}$-norm penalty method, which means that the proposed method exhibits higher resolution than both of them.

**Figure 3:**
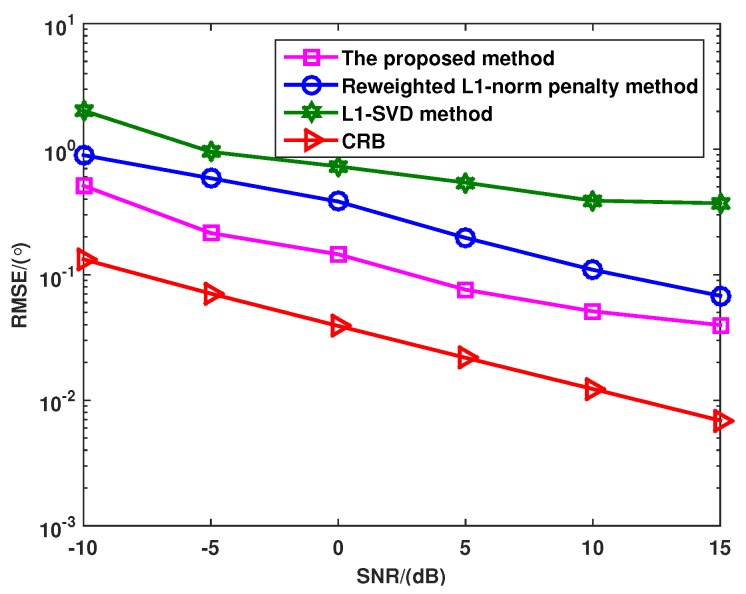
RMSE of the $l_{1}$-SVD method, the reweighted $l_{1}$-norm penalty method and the proposed method when the SNR varies from −10 dB to 15 dB.

Figure 3 shows the RMSE *versus* SNR with different methods, where there are P=3 uncorrelated targets with the DOAs as $\theta_{1}=-8$°, $\theta_{2}=0$° and $\theta_{3}=10$°, and the number of snapshots is 100. As can be seen from Figure 3, the reweighted $l_{1}$-norm penalty method has a lower RMSE than the $l_{1}$-SVD algorithm in all SNR region. This is because the reweighted $l_{1}$-norm penalty method uses the capon spectrum to design the weight matrix for enforcing the sparsity of the solution. On the other hand, the proposed method outperforms both the $l_{1}$-SVD algorithm and the reweighted $l_{1}$-norm penalty method at all SNR regions, and the RMSE of the proposed method is closer to CRB than other methods. The reason is that both the noncircularity of signals and the reweighted $l_{1}$ norm penalty are used to enhance the sparsity of the solution in the proposed method.

**Figure 4:**
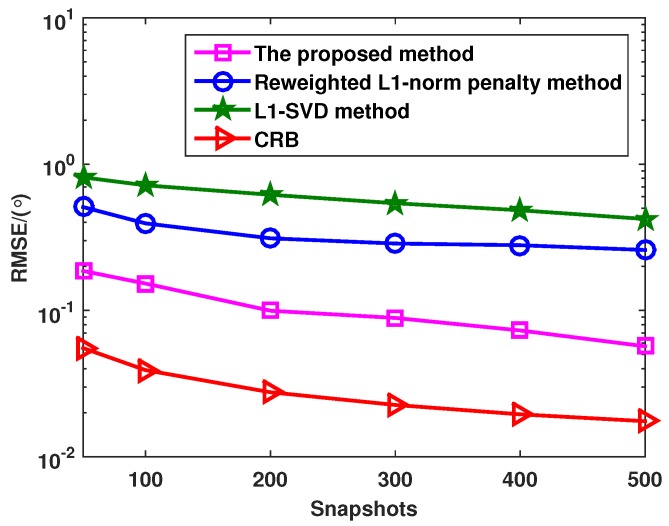
RMSE of the $l_{1}$-SVD method, the reweighted $l_{1}$-norm penalty method and the proposed method when the snapshots varies from 50 to 500.

Figure 4 shows the RMSE *versus* snapshots with different methods, where there are P=3 uncorrelated targets with the DOAs as $\theta_{1}=-8$°, $\theta_{2}=0$° and $\theta_{3}=10$°, and the SNR is fixed at 0 dB. From Figure 4, it can be seen that the angle estimation performance of all methods is improved with the increased snapshots. Furthermore, the proposed method provides better performance than the $l_{1}$-SVD and the reweighted $l_{1}$-norm penalty methods.

**Figure 5:**
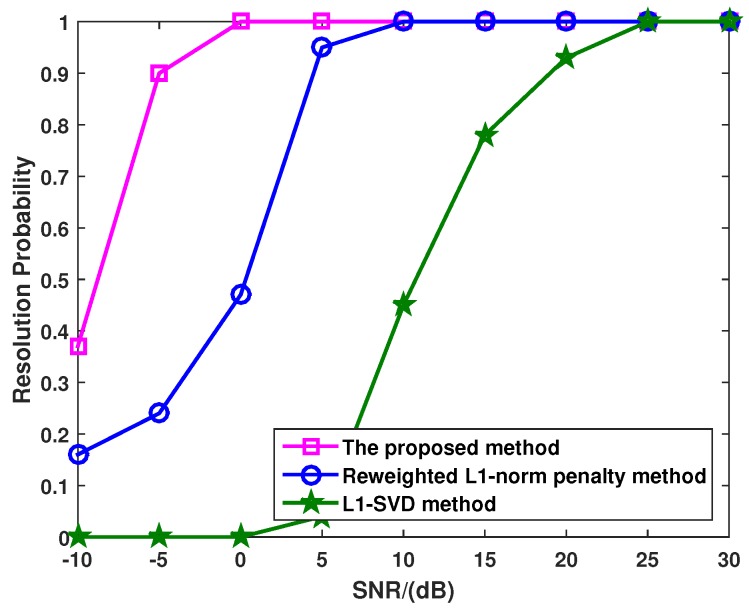
Target resolution probability of the $l_{1}$-SVD method, the reweighted $l_{1}$-norm penalty method and the proposed method when the SNR varies from −10 dB to 30 dB.

Figure 5 shows the target resolution probability *versus* SNR with different method, where there are P=3 uncorrelated targets with the DOAs as $\theta_{1}=-8$°, $\theta_{2}=0$° and $\theta_{3}=10$°, and the snapshots are fixed at 100. In this simulation, all targets can be seen as successful detections when the absolute DOAs for all targets are within 0.5°. As seen in Figure 5, all methods achieve 100% successful detection probability when the SNR is high enough. On the other hand, the probability of target resolution for each method begins to descend at a certain point, which is known as the SNR threshold. Both the $l_{1}$-SVD and the reweighted $l_{1}$-norm penalty methods provide higher SNR threshold than the proposed method, which means that the proposed method has superior angular resolution when detecting closely-spaced targets.

**Figure 6:**
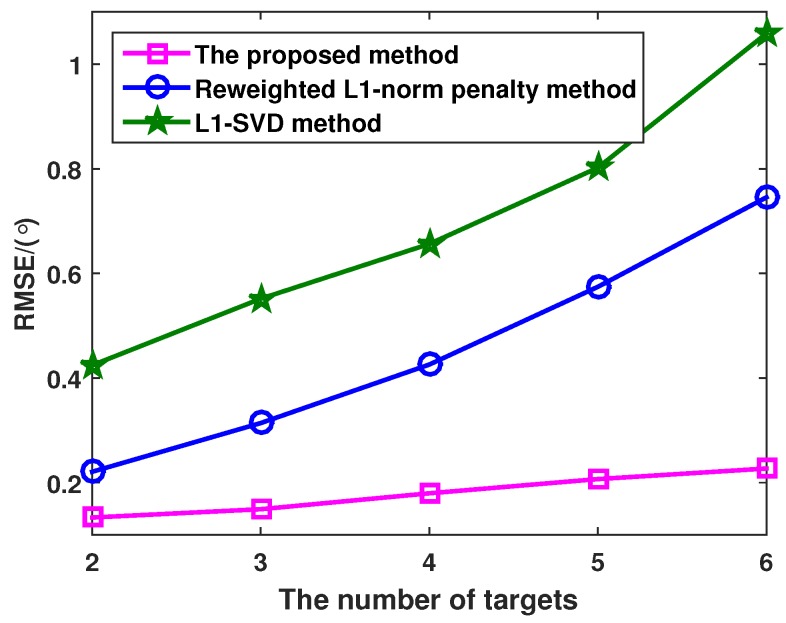
RMSE of the $l_{1}$-SVD method, the reweighted $l_{1}$-norm penalty method and the proposed method *versus* the number of targets.

Figure 6 shows that RMSE *versus* the number of targets with different methods, where the SNR and the number of snapshots are set as 0 dB and 100, respectively. Assuming that the number of targets is *P* and the DOA corresponding to the *p*-th target is -20°+(p-1)10°, from Figure 6, it is indicated that the performance of three methods become poorer with the increased number of targets. In addition, the proposed method exhibits better performance than the $l_{1}$-SVD and the reweighted $l_{1}$-norm penalty methods. This is because the proposed method owns the sparsest solution compared to the $l_{1}$-SVD and the reweighted $l_{1}$-norm penalty methods.

**Figure 7:**
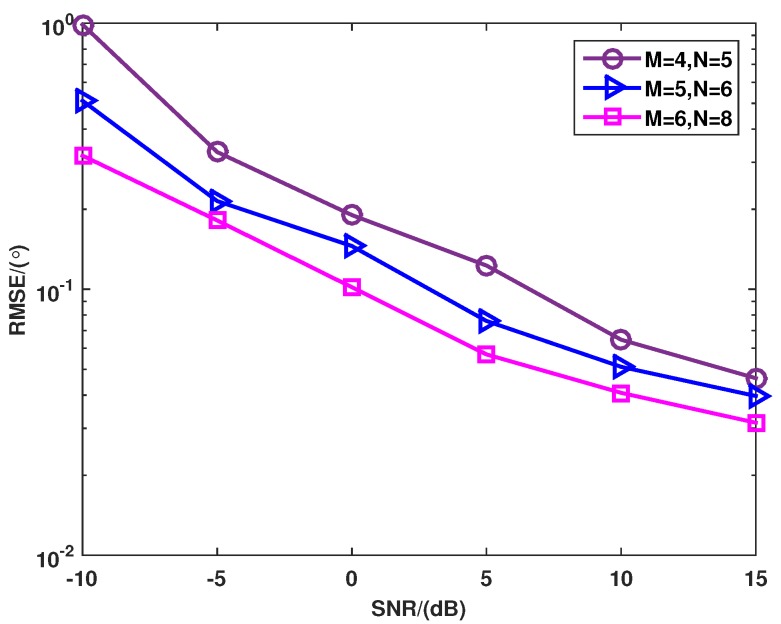
RMSE of the proposed method *versus* different elements when the SNR varies from −10 dB to 15 dB.

Figure 7 shows the RMSE *versus* different transmit and receive elements, where the number of the snapshots is set as 100. The number of targets is P=3, and the DOAs are $\theta_{1}=-8{}^{\circ}$, $\theta_{2}=0{}^{\circ}$ and $\theta_{3}=10{}^{\circ}$, respectively. As the number of transmit and receive elements increases, the performance of the proposed method can be improved. This is because the more transmit and received elements the MIMO radar has, the more spatial diversity gain can be achieved.

## 6. Conclusions

In this paper, we proposed a novel sparsity-aware DOA estimation scheme for a noncircular source in MIMO radar. The proposed method exploits the noncircularity of signals and the weight matrix to formulate the joint sparsity-aware scheme for enhancing the sparsity of solution, which improves the DOA estimation performance. Simulation results verify that compared to the $l_{1}$-SVD and the reweighted $l_{1}$-norm penalty methods, the proposed method achieves better angle estimation performance and higher resolution.
